# Pharmacokinetics of oral Δ9-tetrahydrocannabinol (THC) administration in vervet monkeys

**DOI:** 10.1186/s42238-026-00422-y

**Published:** 2026-03-24

**Authors:** Frédéric Huppé-Gourgues, Natalie Spridzans, Simon G. Lamarre, Amy Beierschmitt, Roberta M. Palmour, Jean-François Bouchard, Maurice Ptito

**Affiliations:** 1 École de psychologie, Université de Moncton, 18 Antonine-Maillet, Moncton, New-Brunswick E1A 3E9 Canada; 2 Dépt Chimie et Biochimie, Université de Moncton, New-Brunswick, Canada; 3 Département de Biologie, Université de Moncton, New-Brunswick, Canada; 4 Behavioral Science Foundation of St-Kitts, West Indies, Saint Kitts and Nevis; 5 Faculty of Medicine and Health Sciences, McGill University, Montréal, QC Canada; 6 École d’Optométrie, Université de Montréal, Montréal, Québec Canada; 7 Department of Applied Mathematics and Computer Sciences, Technical University of Denmark, Lyngby, Denmark; 8 Institute of Neuroscience (IoNS), Faculté de Médecine, Université Catholique de Louvain, Bruxelles, Belgique

## Abstract

Cannabis and its primary psychoactive component, Δ9-tetrahydrocannabinol (THC), are increasingly studied for their pharmacological effects, yet little is known about their pharmacokinetics in non-human primates. This study aimed to characterize the pharmacokinetic profile of THC and its metabolites following oral administration in vervet monkeys under both acute and short chronic exposure conditions. Using high-performance liquid chromatography-tandem mass spectrometry (HPLC-MS/MS), we quantified delta 9-THC, 11-hydroxy-THC (11-OH-THC), and 11-nor-9-carboxy-THC (THC-COOH) in blood samples. THC concentrations increased after dosing, with peak values observed around 4 h post-ingestion. On day 1 and day 8, the 4-hour increase was observed across dose groups. The baseline (0 h) THC on Day 8 was increased when compared to Day 1. 11-OH-THC showed a similar peak around 4 h time course. CBD was increased only at day 1 for the highest dose and THC-COOH was not significantly increased. These findings provide a pharmacokinetic foundation for future research on THC’s therapeutic and behavioral effects in vervet monkeys and help inform dosing strategies for translational studies.

**Supplementary Information:**

The online version contains supplementary material available at 10.1186/s42238-026-00422-y.

## Introduction

Cannabis (from *C. sativa* and *C. indica*) has continued to increase in popularity, with one in six Canadians reporting its use within the last 30 days (Health Canada, 2024). Cannabis is often used for the purpose of getting “high”, via the primary psychoactive constituent, Δ9-tetrahydrocannabinol (THC) (Turner et al. 1980), through varying routes of administration (Spindle et al. 2019). Cannabis use is classified into recreational, medical, occasional, or chronic habits (Health Canada, 2024) depending on dose, frequency, and the reason why it is consumed. Cannabis is widely available, frequently used, and has powerful psychoactive effects (Downey and Verster 2014; Hall and Degenhardt 2015). It is also the most commonly detected drug after alcohol in fatal vehicle collisions (Woodall et al. 2015).

Most studies have focused on the effects of smoked cannabis on various cognitive and socio-affective behaviors. Some have reported poorer cognitive functions that lead to deficits in learning and memory, attention, and working memory (Crean et al., 2011; Crean et al. 2011; Ranganathan and D’Souza 2006), while others have found planning problems, slower brain-processing power, difficulties in motor skills and language and in exercising inhibition (Grant et al. 2018; Pillay et al. 2008; Rodríguez de Fonseca et al., 2005; Schreiner and Dunn 2012; Solowij and Pesa 2012). At the psychological level, THC consumption has been associated with a higher risk for neuropsychiatric diseases, including mood disorders, substance abuse, and schizophrenia (Volkow et al. 2016). It is important, however, to acknowledge the relevance and unique challenges associated with oral doses (Zhao et al. 2024). Few studies have been conducted on edibles, although consuming cannabis orally is the second most popular route of administration (Health Canada, 2024).

Despite the widespread use of recreational cannabis, only 31 medicinal human clinical trials have been registered in Canada (Di Ciano et al. 2024) since legalization in 2018 (Rosenberg 2024), and few studies have determined the long-term observed behavioral effects in chronic users. The pharmacokinetics of THC and its metabolites have been established in humans (Huestis et al. 1992) and are commonly analyzed by LC-MS/MS (Schwope, Scheidweiler, et al., 2011; Scheidweiler et al. 2016). Many of these studies quantified THC and phase I and II metabolites (Schwope et al. 2011a, b; Schwope, Scheidweiler, et al., 2011), often in occasional cannabis users, via various routes of administration (Newmeyer et al. 2016).

Although the human pharmacokinetics of ${{\Delta}}^{9}$-tetrahydrocannabinol (THC) are relatively well characterized under controlled experimental conditions (Baglot et al. 2021), animal models that accurately reflect human consumption patterns remain critical for studying the impact of cannabis on brain and behavior. Most animal studies to date utilize THC injections, which produce physiological and behavioral effects markedly different from inhalation or oral ingestion, likely due to distinctive pharmacokinetic profiles. For instance, Baglot et al. (2021) demonstrated that even when peak plasma THC concentrations are matched, the route of administration—such as intraperitoneal injection versus inhalation—can generate distinct time-courses for THC and its active metabolite 11-OH-THC in both plasma and brain. However, while inhalation models are increasingly common, oral administration remains significantly under-studied in the preclinical literature, despite being a prevalent and growing route of human consumption through ‘edibles’ and oils. This gap is further complicated by evidence from large-animal work in pigs, which has shown that THC distribution to peripheral tissues is highly species-dependent (Brunet et al. 2006). Consequently, there is a clear need for pharmacokinetic data in large-animal models following oral exposure to better justify translational research and improve data interpretation across species (Baglot et al. 2021; Liu and Martin 2018 for review).

Despite well-characterized human THC pharmacokinetics under controlled conditions, critical gaps remain for chronic users (approximately 10% of all users; World Health Organization, 2016), and human observational studies are confounded by polydrug use, as cannabis is frequently consumed with tobacco and alcohol. These limitations further underscore the need for controlled non-human primate studies to isolate THC pharmacokinetics from external variables.

In non‑human primates, cannabinoids have predominantly been studied from a behavioural and dependence perspective. Squirrel monkey, rhesus and cynomolgus studies showed that THC and other CB₁ receptor agonists can function as reinforcers, produce tolerance and dependence, and interact with other drugs of abuse (John et al. 2017; Justinova et al. 2013; McMahon 2011). More recently, inhalation models in rhesus macaques have demonstrated self‑administration of aerosolized THC and synthetic cannabinoids using an ecologically relevant pulmonary route (Cooper et al. 2021). However, these studies have rarely included detailed characterization of THC and metabolite pharmacokinetics, and blood THC levels do not consistently predict behavioural or physiological effects in primates with differing exposure histories (Ginsburg et al. 2014). Consequently, there is a paucity of systematic pharmacokinetic data for THC in non‑human primates, particularly under dosing regimens and routes that mirror human cannabis use. Beyond these pharmacokinetic gaps, monkeys offer closer homology to humans than rodents in brain organization, endocrine function and cannabinoid‑modulated cognitive and affective behaviours (John et al. 2017), yet current dosing strategies for non‑human primate THC studies are often guided by human or rodent data in the absence of species‑specific pharmacokinetic information.

Considering the ethical imperative to minimize and refine animal use, establishing robust pharmacokinetic profiles in a translationally relevant primate model is a critical prerequisite for large-scale studies of chronic cannabinoid exposure. To address this, the present study aims to quantify THC and its primary metabolites in the whole blood of vervet monkeys (*Chlorocebus aethiops sabaeus*) following oral ingestion of various doses, using high-performance liquid chromatography-mass spectrometry. By defining these profiles through a human-relevant route of administration, this work provides essential baseline data for rational dose selection and the interpretation of exposure–effect relationships. Ultimately, these findings enhance the translational value of the non-human primate model, offering a highly controlled framework that complements clinical research in human participants.

## Materials and methods

### Dose administration and sample collection

Twenty-four adolescent vervet monkeys (*Chlorocebus aethiops sabeus*) were divided into four groups of six each. Each group comprised females (*n* = 2) and males (*n* = 4), approximately 4 years of age. The animals weighed between 3 and 4.5 kg with similar body condition score of 2.5-3 which correspond to lean to optimal health range (Clingerman and Summers 2012). All animals were bred on the premises of the Behavioral Science Foundation of St-Kitts (West Indies) following an approved ethics protocol by animal care committee. None of the animals had prior exposure to cannabinoids and all of them were drug- and experiment-naive. For the duration of the study, animals were individually housed with *ad libitum* access to food and water. Individual cages were outdoors and exposed to natural light/dark cycles. The cages were sufficiently close to allow auditory, olfactory, and visual interactions with the local environment and conspecifics. The cages had mirrors or other enrichment device, perches and food enrichment was provided daily. The experimental protocol was reviewed and approved by the local Animal Care and Use Committee (University of Montreal, protocol # 14 − 007) and the Institutional Review Board of the Behavioral Science Foundation that is recognized by the Canadian Council on Animal Care (CCAC). All efforts were made to minimize stress and enhance the experience of the animals. Blood import permit from St. Kitts to Moncton was obtained from the Canadian Food Inspection Agency.

THC was obtained by the National Institute of Drug Abuse (NIDA, USA, Lot #B1150-161204 diluted in ethanol: Δ9-THC > 99.5%, CBD < 0.05% concentrations). The experimental session lasted for 8 days, with samples taken on days 1 and 8. The 24 subjects were randomly assigned to a dose to be administered on days 1 and 8: Group A: 0.5 mg/kg, Group B: 1 mg/kg, Group C: 2 mg/kg and Group D: 3 mg/kg. Appropriate maintenance doses were provided on Days 2–7; for example, an animal in the 2 mg/kg group weighing 3 kg received 6 mg of THC infused into a piece of banana. On Day 0, each monkey was anesthetized with ketamine (dose based on weight) for a transfer to an individual cage, where it remained during the experiment. The weight of each animal was recorded, and a 2 mL femoral blood sample was taken as a baseline control sample. Blood was preserved with anticoagulant ethylenediamine tetraacetic acid (EDTA) and frozen. The day before day 1 and day 8 all animals were overnight fasted. Around 9 a.m. on day 1 each animal received an appropriate dose of THC by oral gavage while under ketamine sedation (5 mg/kg intramuscular). To ensure safe handling, additional ketamine was administered as needed prior to subsequent blood draws until the 8th hour post-THC. The next day ketamine was administered for the 24 h blood collection. Femoral blood samples were taken at 0 h, 0.25 h, 0.5 h, 1 h, 2 h, 4 h, 8 h, and 24 h. The animalswere monitored every 0.5 h to avoid adverse dose-related effects. From Days 2 to 7, the animals were offered a piece of banana infused with the THC in alcohol solution (total volume 150 µl), and consumption was monitored to ensure that the entire piece was eaten. All animals readily and voluntarily consumed the bait. During these days, there was no blood sampling or anesthesia. On Day 8, after the 24 h blood sample, the animals were weighed and returned to their usual group housing. Throughout the study, no changes in body weight, no abnormal behaviors, and no other adverse effects was observed by the attending veterinarian. The samples were frozen and shipped on dry ice.

### Software, reagents, and HPLC-MS/MS parameters

Sample preparation and analysis followed the Agilent forensic protocol for cannabinoid detection in human blood, including solid phase extraction using Agilent Technologies’ EMR-Lipid columns as described elsewhere (Stevens et al. 2020). Standards were supplied by Supelco, including (–)-trans-Δ9-THC, (–)-Δ9-THC-D3, cannabidiol, cannabidiol-D3, (±)-11-hydroxy-Δ9-THC, (±)-11-hydroxy-Δ9-THC-D3, (±)-11-nor-9-carboxy-Δ9-THC, and (±)-11-nor-9-carboxy-Δ9-THC-D3. Analyte separation and quantification were performed using an Agilent 1100 HPLC system coupled to a triple quadrupole mass spectrometer (Agilent Ultivo G6465B). Separation was achieved using a C18 column (Agilent InfinityLab Poroshell 120 EC-C18 2.1 × 100 mm, 2.7 μm), maintained at 38.0 °C with a flow rate of 0.4 mL/min. The mobile phases were (A) water with 0.1% formic acid and (B) acetonitrile with 0.1% formic acid. The elution gradient started at 40% A (60% B) and was held for 1 min, then linearly increased to 77% B by 7 min, followed by a ramp to 95% B at 8.2 min. The system was then returned to initial conditions, with a total run time of 15 min. The autosampler was held at 5 °C with a 5 µL injection volume. Electrospray ionization (ESI) in positive mode was used, with a gas temperature of 350 °C at a flow rate of 10.0 L/min. For each analyte, two transitions were detected (Supplementary Table 1). Calibration standards ranged from 0.06 to 1000 ng/mL and were fitted using weighted (1/x) linear regression. Concentrations were determined by back-calculation from the calibration curve. Quality control (QC) samples analyzed with each batch showed a mean accuracy of ~ 95% across all levels. External calibration curves and QC samples were prepared in rat blood matrix. Quantification was carried out using MassHunter Quantitative Analysis (version 10.2) by integrating the peak area of each analyte, normalizing to its corresponding deuterated internal standard, and determining concentrations by interpolation from the calibration curve. The empirical limit of detection (LOD) was 0.18 ng/mL, defined by a signal-to-noise ratio of 3.6 for 11-OH-THC, 2.4 for THC_COOH and 4.5 for THC, respectively. Concentrations near this empirical detection limit were interpreted qualitatively rather than used for quantitative pharmacokinetic modelling.

Outliers, defined as values greater than three standard deviations from the mean, were removed from the sample set. In cases where the removal of outliers rendered statistical analysis impossible, the data were not reported. We used a mixed-effects model analysis for repeated measures with two factors: an intrasubject factor (time) and an intersubject factor (doses) (SAS 9.4). Analyses were conducted separately by day. When a significant effect was detected, post hoc tests with Tukey–Kramer adjustment were performed. Analyses were conducted after a base-10 logarithmic transformation of the data to address heteroscedasticity. The normality assumption was assessed using a Q–Q plot of the residuals.

## Results

The median of plasma concentrations (with sample size) for THC, 11‑OH‑THC, THC‑COOH, and CBD at baseline (0 h), 4 h, and 24 h on Day 1 (acute exposure) and Day 8 (end of short chronic administration) are presented in Supplementary Table 2.

### Day 1 (acute exposure)

**Fig. 1:**
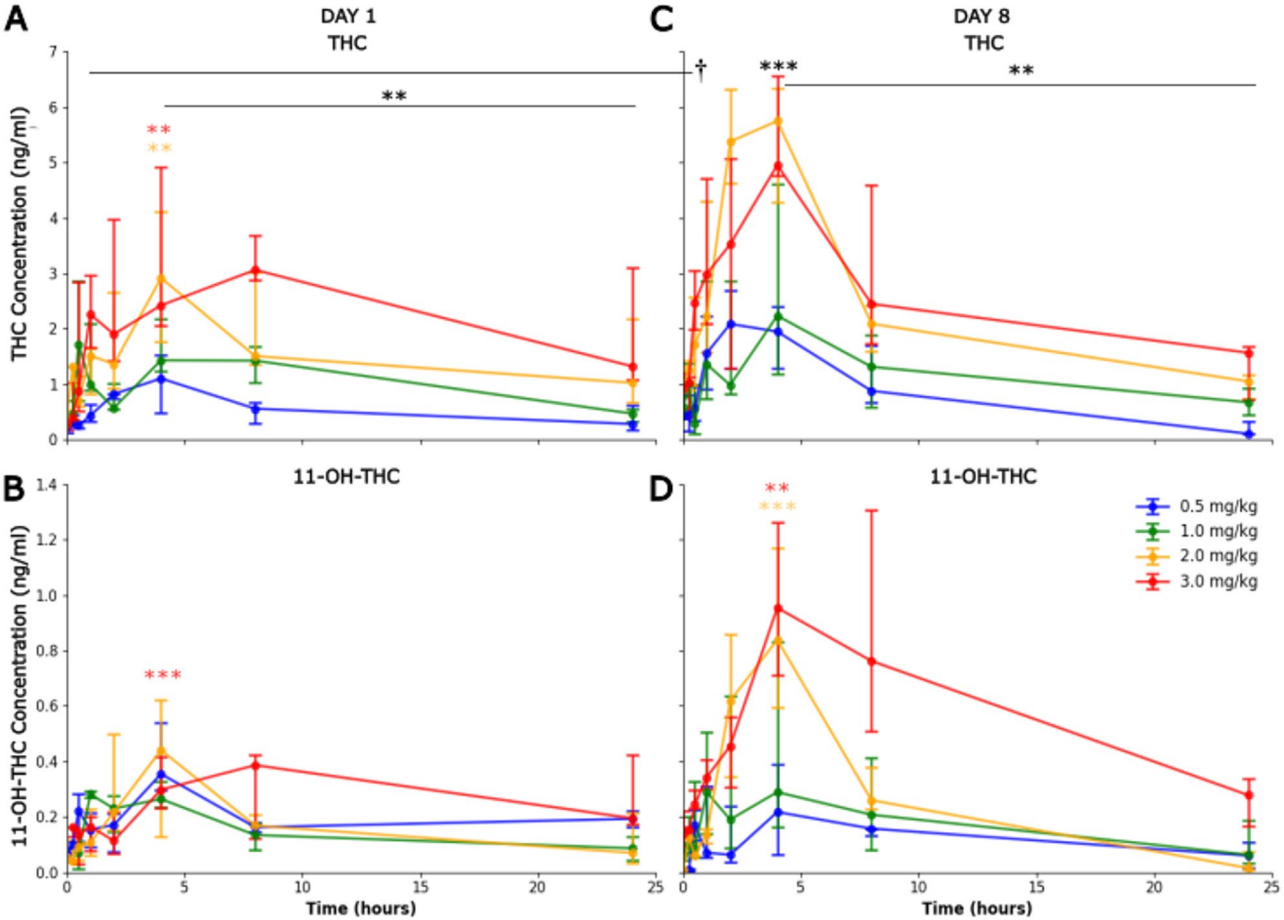
Plasma THC and 11-OH-THC concentrations following oral administration in vervet monkeys on Day 1 (acute) and Day 8 (short-chronic exposure). Blood THC (A, C) and 11-OH-THC (B, D) concentrations measured over 24 h following oral THC administration in adolescent vervet monkeys (*Chlorocebus aethiops sabeus*; *n* = 6 per dose group; 4 males, 2 females per group). Four dose groups received 0.5, 1.0, 2.0, or 3.0 mg/kg THC by oral gavage. Left panels (**A**, **B**): Day 1 acute exposure. Right panels (**C**, **D**): Day 8 following daily dosing (Days 1–8). Data represent median ± interquartile interval (IQI). Outliers (> 3 SD from mean) were excluded. Statistical analysis: mixed-effects model for repeated measures with time (intrasubject) and dose (intersubject) as factors, analyzed separately by day; post hoc Tukey-Kramer adjustment. Black indicates main effect, color code indicates specific effect *p < 0.05, **p < 0.01, ***p < 0.001; † *p* < 0.05 vs. Day 1 baseline for Day 8 comparisons

THC different administered doses showed a main effect on the measured level of THC in the blood (F_3, 19_=6.98, *p* = 0.002), the time course also showed an effect (F_7, 119_=14.18, *p* < 0.001), and there was no interaction. The increase was observed with doses of 2 mg/kg (β = 0.452, *p* = 0.007) and 3 mg/kg (β = 0.517, *p* = 0.003). The β coefficient represented the fixed-effect estimate, indicating the magnitude and direction of the association between the predictors and the dependent variable. Across doses THC increased from baseline at all the further time points after 30 min post administration. Most importantly, the 4 h post-dose timepoint (all doses) corresponded to T_max (β = 0.757, *p* < 0.001) consistent with the expected time course. THC concentrations then decreased until 24 h (β=-0.335, *p* = 0.007), at which point they had declined toward baseline levels (Fig. 1A).

For 11‑OH‑THC, no main effect was observe on Day 1 for the dose (F_3, 18_=0.21, *p* = 0.887), but a main effect was measured for the time course (F_5, 67_=4.01, *p* = 0.003), only the higher concentrations were statistically significant at 4 h (β = 0.713, *p* = 0.001) (Fig. 1B). THC‑COOH did not show a significant increase at 4 h compared with baseline on Day 1 (F_3, 20_=0.58, *p* = 0.635). CBD showed a statistically significant increase on Day 1 (F_3, 17_=5.58, *p* = 0.008) only significantly at the higher concentration 3 mg/kg (β = 0.192, *p* = 0.01).

### Day 8 (end of short chronic administration)

On Day 8, THC again showed a dose effect (F_3, 20_=3.61, *p* = 0.031) with the 4 h corresponding T_max (F_7, 138_=10.59, *p* < 0.001), followed by a decline during the next 24 h (β=-0.722, *p* < 0.001) (Fig. 1C).

11‑OH‑THC showed an interaction effect of the time with the dose on Day 8 (F_5,78_=2.63, *p* = 0.003), with higher concentrations peaked at 4 h 2 mg/kg (β = 1.20, *p* < 0.001) and 3 mg/kg (β = 0.703, *p* = 0.005) (Fig. 1D). THC‑COOH and CBD were not significantly different from baseline at 4 h on Day 8, and neither analyse showed evidence of a consistent time-dependent shift over the sampling window.

### Day 1 vs. day 8 baseline (0 h) comparison

Baseline (0 h) THC concentrations significantly increased from Day 1 to Day 8 (β = 0.419, *p* < 0.001). This accumulation was most pronounced at higher doses; specifically, median baseline values at 2 mg/kg rose from 0.39 ng/ml on Day 1 to 0.934 ng/ml on Day 8 (a 2.4-fold increase), while at 3 mg/kg, values rose from 0.275 ng/ml to 0.936 ng/ml (a 3.4-fold increase).

## Discussion

The present study aimed to quantify THC and its principal metabolites under both acute and short chronic administration in vervet monkeys, providing an initial pharmacokinetic profile to guide future research in this species. Consistent with expectations and prior research on oral cannabis in humans (Spindle et al. 2020), we observed clear dose-dependent increases in THC, with peak concentrations occurring around 4 h post-ingestion. This timing aligns well with the slower absorption phase associated with oral administration of cannabinoids (Vandrey et al. 2017).

### THC and 11-OH-THC

Translational framing: dose selection and cross-species comparison.

The 0.5–3.0 mg/kg edible THC doses used here were selected to produce systemic exposures broadly overlapping with moderate to high human edible intake over a short time frame, rather than to represent a direct mg/kg match to typical human doses. Human oral and edible cannabinoid studies commonly employ single doses of 5–50 mg Δ9‑THC, yielding peak plasma concentrations in the tens of ng/mL range with substantial inter‑individual variability and delayed, sometimes erratic absorption (Spindle et al. 2020; Vandrey et al. 2017; Wall et al. 1983). In addition to the similarity in time to peak, the magnitude of peak THC concentrations in vervets was broadly consistent with those reported in humans receiving comparable oral doses when scaled by body weight and with prior non‑human primate work. In our vervets, oral THC at 0.5–3.0 mg/kg produced median plasma‑equivalent peak concentrations at 4 h (T_max) ranging from 1.11 ng/mL (0.5 mg/kg) to 2.43 ng/mL (3 mg/kg). In rhesus macaques, Perlin et al. (1985) observed C_max values around 40 ng/mL after 2.5 mg/kg oral THC in capsules and ~ 240 ng/mL when the same dose was delivered on a cookie, highlighting the strong influence of formulation on oral bioavailability. Our values are notably lower than those reported by Perlin et al. (1985), being approximately one order of magnitude below their capsule data and nearly two orders of magnitude below their cookie formulation for comparable mg/kg doses. This discrepancy likely reflects a combination of factors including interspecies differences in metabolism, the specific edible formulation used (banana-ethanol bait vs. high-fat cookie), and the potential influence of ketamine sedation on absorption kinetics. While the qualitative pattern of delayed peak concentrations after oral administration is consistent with prior non-human primate work, the absolute C_max values in vervets suggest species- and formulation-specific constraints on oral THC exposure in this model. In rodents, intraperitoneal administration of 2.5 mg/kg THC or inhalation of THC produces plasma concentrations in the range of ~ 50–100 ng/mL with a T_max of ~ 15 min (Baglot et al. 2021), underscoring the added value of non‑human primate models for translational pharmacokinetics when oral or other clinically relevant routes are of primary interest.

### Acute and short-chronic findings in vervets

In acute administration the THC concentration reach a statistically significant increase showing the expected C_max, T_max and later decline. Some lack of significance at intermediary dose is probably due to the very low effective number of subjects. In the short chronic administration, the THC concentration levels peaked around 4 h and declined over the next 24 h. By the final day of the short chronic regimen, detectable THC at time 0 suggested some degree of residual THC, indicative of bioaccumulation, a known effect of THC accumulating in fat tissue (Rawitch et al. 1979). The increase being around three-fold for the two higher doses. However, despite this detectable THC at time 0, concentrations still peaked sharply around 4 h post-administration, confirming the biphasic nature of oral THC absorption and distribution in non-human primates, paralleling findings in human studies (Schwilke et al. 2009).

### Integration with other NHP data and translational implications

Our findings also complement the recent work of Withey et al. (2020), who characterized THC and metabolite concentrations in adolescent squirrel monkeys receiving daily intramuscular THC (1 mg/kg) or THC + CBD. In their study, peak blood THC concentrations of 44 ng/mL were observed 3 h after intramuscular administration and remained relatively stable over 4 months of daily dosing, with 24 h post‑dose THC levels of 8 ng/mL and brain concentrations approximately twofold higher than blood. In our vervets, oral dosing produced lower and more delayed peaks than intramuscular administration in Withey et al. (2020), as expected from route‑dependent bioavailability, yet detectable residual THC at time 0 on the final day of our short‑chronic regimen is consistent with the persistence of THC in blood and brain reported in that study. Together, these data indicate that adolescent non‑human primates exhibit prolonged systemic and central exposure to THC under both parenteral and oral dosing regimens, and that the vervet edible model extends this work to a clinically relevant oral route.

### THC-COOH

The secondary and inactive metabolite THC-COOH was detected later in the time course, consistent with its slower formation and longer elimination half-life noted in prior studies (Vandrey et al. 2017). In both the acute and short chronic phases, THC-COOH did not reach levels significantly higher than the baseline. Importantly, THC-COOH reference standards were consistently quantified with the selected MRM transition, confirming that the absence of THC-COOH in biological samples reflects a true lack of metabolite formation rather than an analytical limitation. This may be because our sampling points did not fully capture the maximal accumulation of THC-COOH, or because the oral doses and the window of observation did not yield pronounced increases above the baseline. Further investigations involving extended sampling windows may be necessary to clarify the trajectory of THC-COOH accumulation and elimination in this species.

### CBD levels

The CBD levels only rise significantly above baseline for the highest dose and only for the acute administrations. One possible explanation could be the relatively small proportion of CBD present in the administered formulation (< 0.05%), resulting in minimal CBD absorption. Alternatively, metabolic competition with THC or other cannabinoids, as well as differences in first-pass metabolism, might have limited observable increases in CBD. Future studies that control for CBD dose or use formulations with a higher CBD: THC ratio could help clarify CBD’s distinct pharmacokinetic profile in vervet monkeys.

Taken together with prior work, these data help position the vervet edible model within the broader pharmacokinetic literature. In rhesus monkeys, Perlin et al. (1985) showed that oral THC in gelatin capsules exhibited low and variable bioavailability with delayed and highly variable time to peak, whereas intramuscular formulations produce more rapid and predictable exposure. Consistent with this, our oral regimen produced substantially lower peak blood THC concentrations (e.g., ~ 1.1–2.4 ng/mL at 4 h post-dose) than those reported after intramuscular dosing in adolescent squirrel monkeys (44 ng/mL at 3 h post-dose following 1 mg/kg i.m.; Withey et al. 2020). This difference is expected given that oral THC bioavailability in humans is typically low and variable, ranging from 4% to 12% due to extensive first-pass metabolism and gastric degradation (McGilveray 2005; Chayasirisobhon 2020). While specific oral bioavailability in vervets remains to be formally determined, our results align with the well-documented route-dependent differences observed across species (Baglot et al. 2021; Perlin et al. 1985). Within this context, our vervet data add a complementary perspective by characterizing oral THC pharmacokinetics in a non-human primate using an edible formulation that approximates human consumption. The predictable ~ 4 h peak, detectable accumulation across short-chronic dosing, and quantifiable 11-OH-THC and THC-COOH profiles support the utility of this model for designing and interpreting future behavioral and therapeutic studies where oral THC is of interest.

### Limitations

Several limitations must be acknowledged. First, the anesthesia required for blood collection may have influenced the observed pharmacokinetic profile. Ketamine sedation could have contributed to slower gastric transit time, thereby delaying THC absorption (Zholos et al. 2023); if animals’ stomachs were not fully empty despite overnight fasting, absorption may have been further delayed. While not ideal, anesthesia was necessary to allow safe and accurate sampling. More specifically, ketamine itself may have influenced both the gastrointestinal handling and metabolism of THC in this study. Experimental work in mouse small intestine showed that ketamine could suppress muscarinic cation currents and both spontaneous and cholinergic‑induced contractions (Melnyk et al. 2020). More broadly, general anaesthetics including ketamine have been implicated in anaesthesia‑related gut motility disturbances and postoperative ileus via effects on TRP channels and cholinergic signalling (Zholos et al. 2023). Although these data derive from rodents and anaesthetic contexts are distinct from our protocol, they raise the possibility that ketamine sedation during dosing could have contributed to delayed or attenuated THC absorption in vervets. In addition, ketamine and THC likely share overlapping metabolic pathways. Ketamine is primarily N‑demethylated by CYP3A4, CYP2B6 and CYP2C9 in humans (Hijazi and Boulieu 2002; Zanos and Gould 2018), whereas THC is converted to 11‑OH‑THC mainly via CYP2C9 with contributions from CYP3A4 and other CYP2 family isoforms (Huestis 2007; Zendulka et al. 2016). This partial overlap at CYP2C9 and CYP3A4 raises the possibility of competitive metabolism under some conditions, although this has not been empirically tested in non‑human primates or in the dosing paradigm used here. Substrate competition at these enzymes could theoretically affect the rate of 11‑OH‑THC formation and/or THC clearance, potentially blunting peak 11‑OH‑THC concentrations or shifting their timing. Thus, even though our sampling protocol attempted to standardize the timing of anaesthesia relative to dosing, the presence of ketamine means that the exact shape of the concentration–time curves may differ somewhat from unanesthetized oral THC exposure, and this should be borne in mind when extrapolating to awake, freely feeding conditions. Second, the sample size and the limited time points sampled may have reduced our ability to detect subtle differences, especially for metabolites that appear or peak outside the chosen intervals or to calculate statistics in some groups and to compare among sex.

## Conclusion

Overall, this study provides a valuable baseline for understanding the pharmacokinetics of edible THC administration in vervet monkeys during both acute and short chronic exposure. Our findings not only confirm a dose-dependent rise in plasma THC with a predictable peak around 4 h post-ingestion but also highlight the roles of its primary active (11-OH-THC) and inactive (THC-COOH) metabolites. These results offer an important framework for designing future research on the therapeutic or behavioral effects of THC in this non-human primate model, where precise dosing and timing can be critical.

## Supplementary Information

Supplementary Material 1.

Supplementary Material 2.

## Data Availability

The research data supporting the results of this manuscript are publicly available in the Open Science Framework (OSF) repository 10.17605/OSF.IO/5DGJK.
