# Supplementary material for: Pharmacokinetics of oral Δ9-tetrahydrocannabinol (THC) administration in vervet monkeys
Source: J Cannabis Res. 2026 Mar 24;8:60. doi: 10.1186/s42238-026-00422-y (PMC13130606; doi:10.1186/s42238-026-00422-y)
Supplement: Supplementary file 1 — Supplementary Material 1. [file 42238_2026_422_MOESM1_ESM.docx]

**Supplementary** Table 1: MRM acquisition parameters for THC compounds.

| Compound | Precursor ion | Quantifier ion | Qualifier ion | Retention time |
| --- | --- | --- | --- | --- |
| Δ9-THC | 315.2 | 122.3 | 193.0 | 8.67 |
| Δ9-THC-D_3_ | 318.0 | 123.0 | 196.0 | 8.64 |
| 11-hydroxy-Δ9-THC | 331.2 | 313.1 | 193.0 | 3.06 |
| 11-hydroxy-Δ9-THC- D_3_ | 334.2 | 316.1 | 196.1 | 3.04 |
| 11-nor-Δ9-carboxy-THC | 345.2 | 327.1 | 299.1 | 3.22 |
| 11-nor-9-carboxy-Δ9-THC- D_3_ | 348.3 | 330.1 | 302.1 | 3.20 |
| CBD | 315.2 | 123.0 | 193.1 | 5.63 |
| CBD- D_3_ | 318.2 | 123.0 | 196.0 | 5.59 |
