# Supplementary material for: Pharmacokinetics of oral Δ9-tetrahydrocannabinol (THC) administration in vervet monkeys
Source: J Cannabis Res. 2026 Mar 24;8:60. doi: 10.1186/s42238-026-00422-y (PMC13130606; doi:10.1186/s42238-026-00422-y)
Supplement: Supplementary file 2 — Supplementary Material 2. [file 42238_2026_422_MOESM2_ESM.docx]

**Supplementary Table 2: Plasma concentrationby administration doses.**

|  | | | | |  |  |  |
| --- | --- | --- | --- | --- | --- | --- | --- |
|  |  | Day 1 |  |  | Day 8 |  |  |
| **Substance** | **Dose (mg/kg)** | **0h Median (n)** | **4h Median (n)** | **24h Median (n)** | **0h Median (n)** | **4h Median (n)** | **24h Median (n)** |
| THC | 0,5 | 0.241 (6) | 1.107 (5) | 0.362 (5) | 0.439 (6) | 1.953 (6) | 0.110 (5) |
|  | 1 | 0.300 (4) | 1.435 (5) | 0.474 (5) | 0.585 (6) | 2.238 (6) | 0.677 (6) |
|  | 2 | 0.390 (5) | 2.919 (6) | 1.029 (6) | 0.934 (5) | 5.750 (6) | 1.054 (6) |
|  | 3 | 0.275 (4) | 2.426 (6) | 1.326 (5) | 0.936 (6) | 4.955 (6) | 1.570 (6) |
| OH-THC | 0,5 | 0.064 (2) | 0.355 (5) | 0.193 (2) | N/A | 0.218 (5) | 0.061 (4) |
|  | 1 | N/A | 0.264 (5) | 0.087 (2) | 0.065 (4) | 0.289 (5) | 0.063 (6) |
|  | 2 | 0.055 (2) | 0.441 (6) | 0.070 (6) | 0.009 (3) | 0.839 (6) | 0.015 (5) |
|  | 3 | N/A | 0.298 (6) | 0.195 (5) | 0.139 (5) | 0.954 (6) | 0.278 (5) |
| THC-COOH | 0,5 | 0.374 (4) | 0.281 (5) | 0.234 (5) | 0.184 (4) | 0.388 (6) | 0.178 (6) |
|  | 1 | 0.313 (6) | 0.428 (5) | 0.213 (5) | 0.226 (6) | 0.289 (6) | 0.203 (6) |
|  | 2 | 0.306 (5) | 0.272 (6) | 0.316 (5) | 0.210 (6) | 0.333 (5) | 0.226 (5) |
|  | 3 | 0.104 (5) | 0.275 (5) | 0.380 (5) | 0.186 (6) | 0.322 (6) | 0.389 (5) |
| CBD | 0,5 | 0.236 (6) | 0.484 (3) | 0.241 (3) | 0.183 (5) | 0.241 (6) | 0.229 (4) |
|  | 1 | 0.232 (3) | 0.391 (3) | 0.245 (4) | 0.179 (6) | 0.223 (6) | 0.205 (5) |
|  | 2 | 0.272 (3) | 0.332 (6) | 0.422 (4) | 0.269 (4) | 0.229 (5) | 0.231 (5) |
|  | 3 | 0.226 (5) | 0.196 (4) | 0.187 (4) | 0.225 (5) | 0.221 (5) | 0.235 (6) |

ng/ml (number of samples)
